# Lessons learned from contamination with endotoxin originated from the supplement in the cell culture medium

**DOI:** 10.1016/j.reth.2024.03.022

**Published:** 2024-04-03

**Authors:** Koaki Uehara, Eriko Oshiro, Atsushi Ochiai, Ryo Takagi, Masayuki Yamato, Atsunaga Kato

**Affiliations:** a Social Medical Corporation Yuuaikai, Yuuai Medical Center, Advanced Medical Research Center, 50-5, Yone, Tomigusuku-shi, Okinawa 901-0224, Japan; b Institute of Advanced Biomedical Engineering and Science, Tokyo Women's Medical University, TWIns, 8-1 Kawada-cho, Shinjuku-ku, Tokyo 162-8666, Japan

**Keywords:** Endotoxin, Cell-based medicinal products, Cholera toxin, Stratified epithelial cell, Certificate of analysis

## Abstract

**Introduction:**

Endotoxin is a typical pyrogen derived from the outer membrane of Gram-negative bacteria. In fabricating cell-based medicinal products, it is necessary to control endotoxin in the process and the products. In the quality control tests of our clinical study, endotoxin concentration in the culture supernatant of autologous oral mucosal epithelial cell sheets exceeded the criterion value. Therefore, endotoxin measurements were conducted to clarify the cause of the endotoxin contamination.

**Methods:**

The reagents used to prepare the culture medium, the unused culture medium, and the culture supernatants were diluted with pure water. Endotoxin concentrations in the diluted samples were measured.

**Results:**

Endotoxin was detected in both the unused culture medium and the culture supernatant of the epithelial cell sheets at higher concentrations than the criterion value. Therefore, endotoxin concentrations in the reagents used to prepare the culture medium were measured and were found to be below the criterion value, except for cholera toxin. On the other hand, three lots of cholera toxin products were used for the measurement, and the endotoxin concentrations were higher than the criterion value. The results indicate that the endotoxin contamination is caused by the cholera toxin product.

**Conclusions:**

To prevent endotoxin contamination in cell-based medicinal products, endotoxin concentrations in reagents used for the fabrication should be measured in the facility conducting clinical research or confirmed by an adequate certificate of analysis from the manufacturers of the reagents.

## Highlights

• Our clinical study was interrupted by endotoxin contamination.
• The cause of the contamination in the cell-based medicinal product was identified.
• The endotoxin originated from the culture medium supplied with the cholera toxin.
• Cholera toxin products disclosing the amount of endotoxin are available.
• A certificate of analysis should be provided by the material manufacturers.

Endotoxin is a lipopolysaccharide composing the outer membrane of Gram-negative bacteria and a typical pyrogen coursing various biological reactions in infections with Gram-negative bacteria [[1], [2], [3], [4], [5], [6], [7], [8]]. Therefore, bacterial endotoxin tests must be conducted as one of the quality control tests for manufacturing pharmaceuticals, medical devices, and cell-based medicinal products [9]. In our clinical study, oral mucosal cells derived from participants were cultured for fabricating transplantable epithelial cell sheets, which were used as cell-based medicinal products for evaluating the safety of transplanting epithelial cell sheets. As one of the quality control tests for the epithelial cell sheets, the culture supernatants were used for measurements of endotoxin concentration. In one case of the clinical study, the endotoxin concentration was higher than the criterion concentration by which an adequate amount of endotoxin in the cell sheets was determined, therefore, the transplantation of the cell sheets was not conducted in this case. This report presents the background of this clinical study, the causes of the endotoxin contamination in the epithelial cell sheets, and the measures to prevent the contamination based on the results of this study.

Esophageal stenosis is one of the major complications of endoscopic treatment such as submucosal dissection and balloon dilatation. Previous preclinical studies using animal models reveal that esophageal stenosis is prevented by transplantation of autologous epithelial cell sheets prepared by cultivation of stratified epithelial cells such as oral mucosal epithelial cells and epidermal keratinocytes on temperature-responsive cell culture surface [[10], [11], [12]]. Moreover, human autologous oral mucosal epithelial cell sheets have been used for the transplantations onto esophageal ulcers to prevent stenosis after endoscopic dissection to remove esophageal cancer, and the safety of the transplantation has been confirmed in the clinical studies [[13], [14], [15]]. Based on the previous studies, we designed a clinical study to evaluate the safety of transplantation of autologous oral mucosal epithelial cell sheets by which restenosis of the esophagus after balloon dilation would be prevented.

Before the clinical study, we prepared a standard operating procedure (SOP) describing how to fabricate the epithelial cell sheets for use in this study and conducted a validation study using human oral mucosal tissues derived from six volunteer donors to demonstrate that the SOP indicated a reproducible method for fabricating transplantable epithelial cell sheets from oral mucosal tissues. The cell culture media were prepared for each volunteer and participant since autologous serums were used as a culture supplement. In the validation study, the quality control tests, by which the adequacy of the epithelial cell sheets had been determined for transplantations on the esophageal ulcers after endoscopic resections [16], were performed to confirm that the prepared epithelial cell sheets indicated equivalent properties to the cell sheets used in the previous clinical studies. The bacterial endotoxin test was one of the quality control tests and was performed according to Japanese official notation No. 0907-2 issued by Pharmaceuticals and Food Safety Bureau, Ministry of Health, Labour and Welfare of Japan [9]. Based on the previous clinical studies, the acceptance criterion value of endotoxin concentration was set as less than 1.0 EU/mL in the culture supernatants of the epithelial cell sheets [[15], [16], [17]]. The endotoxin concentrations were measured by PTS cartridge FDA 0.01–1.0 EU/mL and Endosafe nexgen PTS (Charles River Laboratories, MA, USA) on the 8th day of the culture process according to the protocol described in the SOP. However, in the validation study, the endotoxin concentration was detected at 6.68 EU/mL in the culture supernatant of oral mucosal epithelial cells derived from Volunteer 04 (Table 1). After this result, oral mucosal cells were prepared from two volunteers (Volunteer 05 and Volunteer 06) and used for the validation study, and the endotoxin concentrations were less than the criterion value (Table 1). Based on the results, we conducted the clinical study, however, endotoxin was also detected at 1.31 EU/mL in the fourth case of the clinical study (Table 1). Therefore, we were not able to continue the clinical study in this case. On the other hand, no Gram-negative bacteria were detected in the culture supernatant by the cultivation of sterility test, suggesting that the origin of the endotoxin was assumed to be derived from the reagents used as materials for preparing the culture medium. Then, we conducted endotoxin measurements in the reagents and the culture media to clarify the cause of the endotoxin contamination in the culture supernatant derived from the epithelial cell sheets.

**Table 1:** Endotoxin concentrations in supernatants from cultivation of human oral mucosal epithelial cells.

Sample ID	Sampling day	Dilution factor	Endotoxin conc. (EU/mL)
Validation study			
Volunteer 01	Day 8	50	UD
Volunteer 02	Day 8	50	UD
Volunteer 03	Day 8	50	0.57
Volunteer 04	Day 8	50	6.68
Volunteer 05	Day 8	50	0.90
	Day 14	50	0.88
	Day 16	50	0.65
Volunteer 06	Day 8	50	0.56
Clinical study			
Participant 01	Day 8	50	UD
Participant 02	Day 8	50	0.60
Participant 03	Day 8	50	0.93
Participant 04	Day 8	50	1.31

UD: measurement value was under the low detection limit (0.01 EU/mL) of the endotoxin measurement.

To ensure the accuracy of our measurement of endotoxin concentrations, we outsourced an endotoxin measurement in the culture supernatant of the epithelial cells derived from Participant 04 to a third-party organization (Biomedica Solution Co., Ltd. Osaka, Japan). The measurement was performed by a turbidimetric method, and endotoxin was detected at 1.74 EU/mL in the supernatant. Next, the endotoxin measurements were conducted by using cryopreserved culture supernatants, and the endotoxin concentrations in the supernatants derived from Volunteer 04 and Participant 04 were higher than the criterion value. Moreover, the endotoxin concentrations in the cryopreserved supernatants derived from Volunteers 01, 02, 03, 05, 06 and Participants 01, 02, 03 were also less than the criterion value, suggesting that the endotoxin concentrations of these culture supernatants are not false negative.

To determine whether the endotoxin contamination was caused during the operation for fabricating the epithelial cell sheets, the endotoxin concentration in the unused fresh culture medium prepared for this case was measured and detected at 2.97 EU/mL in the medium (Table 4). As mentioned above, the sterility test detected no contamination with any bacteria in the culture supernatant. Therefore, these results indicate that the detections of endotoxin originate from the unused fresh culture medium rather than from Gram-negative bacteria in the culture supernatant of the cell sheets. To identify the reason for the endotoxin contamination, we measured the endotoxin concentrations in the reagents used as materials for preparing the culture medium. Information about the reagents is shown in Table 2. Of these reagents, there was no cholera toxin sample for the endotoxin measurement, because the cholera toxin had been all used up by preparing the culture medium. The endotoxin concentrations in the reagents, except cholera toxin, are shown in Table 3. Among the results, the amount of endotoxin in EGF is shown by the certificate of analysis from the manufacturer, since the EGF is manufactured as a good manufacturing practice product. These endotoxin measurements detected no endotoxin in concentrations high enough to affect the culture medium after preparation, indicating that the reagents excluding cholera toxin were not the source of endotoxin detected in the culture supernatant of the epithelial cell derived from Participant 04.

**Table 2:** Information about the reagents used as materials for the culture media.

Reagents	Abbreviations	Manufacturer	Cat. No.	Final conc.
Dulbecco's Modified Eagle's Medium	DMEM	SIGMA-ALDRICH	D6546	75 (v/v)%
Nutrient Mixture F-12 HAM	F-12	SIGMA-ALDRICH	N4888	25 (v/v)%
L-glutamine solution	L-gultamin	SIGMA-ALDRICH	G7513	0.16 mM
100 units/ml Humulin R Injection	Insulin	Nippon Eli Lilly	–	4.94 μg/mL
Solu-Cortef Injection 100 mg	Hydrocortisone	Pfizer	–	0.4 μg/mL
hEGF recombinant	EGF	HIGETA SHOYU	REG100UG	10 ng/mL
Cholera toxin solution	Cholera toxin	FUJIFILM Wako Chemicals	030-20621	84 ng/mL
3,3′,5-triiodo-L-thronine sodium salt	Triiodothyronine	MP Biomedicals	194585	1.35 ng/mL
Gentamicin sulfate injection 60 mg “F”	Gentamicin	Fuji Pharmaceutical	–	40 μg/mL
Fungizone Injection	Amphotericin B	Bristol-Myers Squibb	–	0.28 μg/mL

**Table 3:** Endotoxin concentrations in the stock solutions of the reagents used for Participant 04.

Reagents	Stock solutions	Dilution factor	Endotoxin conc. (EU/mL)
DMEM	–	50	UD
F-12	–	10	UD
L-glutamine	10 mM/mL	100	UD
Insulin	3.53 mg/mL	100	UD
Hydrocortisone	10 mg/mL	100	UD
EGF	100 μg/mL	–	<0.01 EU/μg^a^
Cholera Toxin	0.2 mg/mL	Unmeasured	–
Triiodothyronine	13.5 μg/mL	100	UD
Gentamicin	40 mg/mL	100	UD
Amphotericin B	0.5 mg/mL	100	UD

UD: measurement value was under the low detection limit (0.01 EU/mL) of the endotoxin measurement.

a Manufacturer's value of endotoxin test shown in the certificate of analysis.

**Table 4:** Endotoxin in the cholera toxin products.

Manufacturer (Amount of cholera toxin)	Lot	Dilution factor	Endotoxin conc.	
			in the products	in culture media
FUJIFILM Wako Chemicals (100 μg/vial, 200 μg/mL)	CAR3001^a^	300	>300 EU/mL^b^	2.97 EU/mL
	CAM0993	300	>300 EU/mL^b^	4.30 EU/mL
	CAH4664	300	>300 EU/mL^b^	Not implemented
List Biological Laboratories (1 mg/vial)	10069A1	–	12 EU/mg^c^	0.0010 EU/mL^d^
	10068A1	–	45 EU/mg^c^	0.0038 EU/mL^d^
	10067A1	–	5.0 EU/mg^c^	0.00042 EU/mL^d^

a The Same lot of cholera toxin used for cultivation of the oral mucosal epithelial cells derived from Participant 04.

b Estimated value calculated from the results of endotoxin measurement implemented in our facility.

c Manufacturer's value of endotoxin test shown in the certificate of analysis.

d Estimated value calculated from endotoxin test by the manufacturer.

Based on the results of the endotoxin measurements, we measured the endotoxin concentrations in cholera toxin products. We purchased the same lot of the cholera toxin product (Lot No. CAR3001) used for the cultivation of the cells derived from Participant 04 and two different lots of the products (Lot No. CAM0993 and CAH4664) (Table 4). As a result of the measurements, the concentrations in the three lots of cholera toxin were detected above the high detection limit of the endotoxin measurement (Table 4). Among these cholera toxin products, two products (Lot No. CAR3001 and CAM0993) were used to prepare the culture medium according to the preparation procedure, and the endotoxin concentrations in the prepared medium were measured. As a result, the endotoxin concentrations in the culture media were 2.93 and 4.30, respectively, indicating that the concentrations were higher than the criterion value (Table 4). These results indicate that endotoxin contamination in the culture supernatant of the epithelial cell sheets is caused by endotoxin derived from the cholera toxin product used as a culture supplement.

Cholera toxin is available from reagent manufacturers other than FUJIFILM Wako Chemicals. List Biological Laboratories is one of the candidates since a certificate of analysis discloses endotoxin concentrations in cholera toxin supplied by the company. Therefore, the estimated endotoxin concentration in the culture medium supplied with the cholera toxin can be calculated by the certificate. Moreover, the cholera toxin products were used for the fabrication of cultured human oral mucosal epithelial cell sheets in previous clinical studies [13,15]. Therefore, we purchased the cholera toxin manufactured by List Biological Laboratories and reconstituted the procedure for preparing the culture medium supplied with the cholera toxin. The endotoxin concentrations in the cholera toxin products and the estimated endotoxin concentrations in the culture media are shown in Table 4. Among the cholera toxin, one product (lot No. 10069A1) was used for preparing the reconstructed culture medium, and the medium was used for conducting the validation study by using the oral mucosal cells derived from Volunteer 07. As a result, the epithelial cell sheets were successfully fabricated in the culture medium, and no endotoxin exceeding the criterion value was detected in the quality control test during the cultivation (Table 5). Based on the result, we resumed the clinical study in which the cholera toxin used for this validation study (Volunteer 07) was used for preparing the culture medium. The endotoxin measurements in quality control tests of the clinical study for Participant 05 and 06 indicated that the endotoxin concentrations in the unused fresh culture media and the culture supernatants were below the detection limits of the endotoxin test (Table 5).

**Table 5:** Endotoxin concentrations in culture media supplied with cholera toxin manufactured by List Biological Laboratories.

Sample ID	Dilution factor	Endotoxin conc.	
		Fresh culture media	Supernatants at Day 8
Validation study			
Volunteer 07	50	UD	UD
Clinical study			
Participant 05	50	UD	UD
Participant 06	50	UD	UD

UD: measurement value was under the low detection limit (0.01 EU/mL) of the endotoxin measurement.

In the present study, the endotoxin measurements revealed that the cause of endotoxin contamination was the use of the cholera toxin product without a certificate of analysis indicating endotoxin concentration. The cholera toxin products manufactured by List Biological Laboratories were used in the previous clinical studies [13,15]. At the time, because it was easier to purchase cholera toxin products from domestic manufacturers than from foreign ones, we used cholera toxin products manufactured by FUJIFILM Wako Chemicals for our validation and clinical studies. However, the decision was inappropriate from the perspective of the fabrication of cell-based medicinal products and caused endotoxin contamination in the epithelial cell sheets, resulting in the temporary suspension of the clinical study. If the endotoxin concentration in the fresh culture medium had been measured before the cultivation of oral mucosal cells, the contamination with endotoxin derived from the cholera toxin would have been avoided in our clinical study. Indeed, cholera toxin is derived from *Vibrio cholerae* belonging to the Gram-negative bacteria from which endotoxin originates. On the other hand, cholera toxin is one of the useful culture supplements for promoting the proliferation of stratified squamous epithelial cells, such as oral mucosal epithelial cells, by increasing the intracellular concentration of cyclic AMP [[18], [19], [20]]. Indeed, the cholera toxin manufactured by FUJIFILM Wako Chemicals has been used by many researchers as a culture supplement for epithelial cells, and there are no effects on the proliferation and differentiation of human epidermal keratinocytes and keratinocytes differentiated from human embryonic stem cells [21]. Therefore, the concentration of endotoxin derived from cholera toxin by FUJIFILM Wako in the culture medium indicates no effect on the proliferation and differentiation of epithelial cells. Additionally, the quality control tests demonstrate that the endotoxin concentrations in culture media supplied with cholera toxin manufactured from FUJIFILM Wako other than CAR3001 and CAM0993 were less than the criterion value, suggesting the endotoxin concentrations were dependent on the difference of the lot. However, the culture media supplied with a high concentration of endotoxin have been used for preparing *in vitro* models such as wound healing and oral lichen planus, in which the epithelial cells show a decrease in proliferation [[22], [23], [24]]. Basso et al. indicate that viability and viable cell numbers of epithelial cells are reduced by the treatment with lipopolysaccharide derived from *E. coli* [25]. Therefore, a manufacturer of cholera toxin products should be carefully selected to prevent endotoxin contamination in cell-based medicinal products fabricated by the cultivation of stratified epithelial cells.

After the endotoxin contamination in the present study, we selected and used the cholera toxin product manufactured by List Biological Laboratories from which endotoxin concentration in the products was disclosed, successfully fabricating the epithelial cell sheets without endotoxin contamination. Reagents not guaranteed endotoxin concentrations by certificate of analysis from the manufacturers may carry a risk of endotoxin contamination in cell-based medicinal products. Therefore, prior endotoxin measurements should be required when changing lots and/or manufacturers of materials for fabricating cell-based medicinal products. After the present study, we measured the endotoxin concentration in the fresh culture media in every case. These are the lessons learned from the endotoxin contamination in our clinical study.

## Declaration of competing interest

The authors declare that there is no conflict of interest regarding the work described herein.
